# An Innovative Treatment Using Calcium Hydroxyapatite for Non-Surgical Facial Rejuvenation: The Vectorial-Lift Technique

**DOI:** 10.1007/s00266-024-04071-5

**Published:** 2024-05-07

**Authors:** Virginia Marcia Amaral, Helena Hotz Arroyo Ramos, Fernanda Aquino Cavallieri, Mariana Muniz, Guilherme Muzy, Ada Trindade de Almeida

**Affiliations:** 1 IVA Medical Institute, Av. dos Bandeirantes 1518, Belo Horizonte, CEP: 30.315-032 Brazil; 2 Vitória, Brazil; 3 Rio de Janeiro, Brazil; 4 São Paulo, Brazil; 5 São Paulo, Brazil; 6 Hospital do Servidor Público Municipal de São Paulo, São Paulo, Brazil

**Keywords:** Skin aging, Collagen, Plastic surgery, Dermatology, Aesthetics, Calcium hydroxyapatite, Biostimulation

## Abstract

**Background:**

The facial aging process entails alterations in the volume, shape, and texture of all skin layers over time. Calcium hydroxyapatite (CaHA) is a well-established safe skin filler with unique properties to resolve some skin alterations by stimulating neocollagenesis. The vectoral-lift (V-lift) technique targets the global repositioning of facial structures by addressing distinct anatomical injection planes. It includes deep facial augmentation with Radiesse Plus^TM^ to retain ligament restructuring and superficial subcutaneous enhancement with diluted Radiesse Duo^TM^. Herein, we present cases that illustrate the use of this approach.

**Methods:**

This pilot study enrolled 36 participants (33 women and three men; ages 37–68 years) in a Brazilian clinical setting, and all patients underwent a single treatment. Photographs were taken at rest, in frontal and oblique views, before injection, and 90 days after treatment.

**Results:**

Treatment resulted in elevation of the upper and middle face, notable improvements in the infraorbital hollow, and adjustment of the mean facial volume.

**Conclusions:**

The V-lift technique is a three-dimensional pan-facial treatment that relies on ligament support and face vectoring to obtain a lifting effect and facial contour restoration. It encompasses deep facial augmentation involving the use of Radiesse Plus^TM^ for restructuring and retaining ligaments and Radiesse Duo^TM^ for superficial subcutaneous enhancement. This approach targets a global repositioning of the facial structures by addressing distinct anatomical injection planes. It achieves a repositioning of the overall facial anatomy without requiring a substantial volumetric expansion.

**Level of Evidence IV:**

This journal requires that authors assign a level of evidence to each article. For a full description of these Evidence-Based Medicine ratings, please refer to the Table of Contents or the online Instructions to Authors www.springer.com/00266.

## Introduction

Minimally invasive procedures have gained popularity because of their shorter downtime, absence of visible scars, and potential for corrective action if the outcomes deviate from initial goals [1]. Consequently, procedural strategies that account for facial shape, structure, proportions, and the impact of aging on facial esthetics have become focal points of esthetic medicine research [2]. Recent studies have focused on refining the placement of soft tissue fillers on the face, thereby augmenting both results and safety while utilizing less volume of the product for superior and natural final outcomes [3].

It is speculated that the injection of collagen biostimulators around the adhesion areas and retaining ligaments of the face promotes contracture, causing a suspension effect by acting as anchors and, as a result, elevating the tissues and improving the lifting effect [4–6]. This occurs because the facial retaining ligaments serve as pivotal anchoring points for both the superficial musculoaponeurotic system (SMAS) and the overlying dermis onto the deep fascia and periosteum [7].

Moreover, there is a lack of studies that address the physiology and mechanics of non-surgical repositioning of the retaining ligaments of the face with collagen biostimulators [4, 5, 8]. Calcium hydroxyapatite (CaHA) is a well-established and safe dermal filler with unique properties that enhance the mechanical properties of the skin. It stimulates collagen and elastin production, angiogenesis, and the proliferation of dermal cells [9]. However, there is a lack of studies in the literature that address the physiology and mechanics of non-surgical repositioning of retaining ligaments of the face with CaHA injections.

In this study, we aim to introduce a technique that employs CaHA-based dermal filler based on the principles of periligamentar subMAS layer redensification and face vectoring. The technique aims to attain lifting effect and restore facial contours.

## Materials and Methods

### Study Design

This pilot study included 36 participants in a clinical setting. The analysis involved thirty female participants and three males, with an age range of 37–68 years. All patients received a single treatment, and all procedures adhered to the ethical guidelines outlined in the Declaration of Helsinki, as revised in the year 2000.

### Patient Selection and Documentation

For this study, women and men aged 35 years and older were selected if they exhibited midface deflection. Individuals were excluded if they had received hyaluronic acid (HA) fillers, neuromodulators, or biostimulators in the previous 6 months, or if they planned to undergo any other facial enhancement procedures such as fillers, chemical lipolysis injections, energy-based devices, or surgeries in the coming months. Extremely thin patients, those with very heavy faces, or individuals with a surgical indication were also excluded. All participants provided written (signed) informed consent.

Photographs were taken at rest, in frontal and oblique views before calcium hydroxylapatite (CaHA) injection (Day 0) and 90 days after treatment (Day 90), using the same camera (Canon EOS T5i with 50 mm f/1.8 STM lens), lighting, and distance parameters..

### Injection Techniques

To facilitate anatomical subdivision and delineation, the treated regions were categorized as follows: temporal (from the temporal line to the upper edge of the zygomatic arch), zygomatic (covering the zygomatic arch region), masseteric (extending from the lower edge of the zygomatic arch to the masseteric parotid region), orbital, infraorbital, and mandibular regions (spanning from the pre-jowl sulcus area to the angle of the mandible area).

**Fig. 1:**
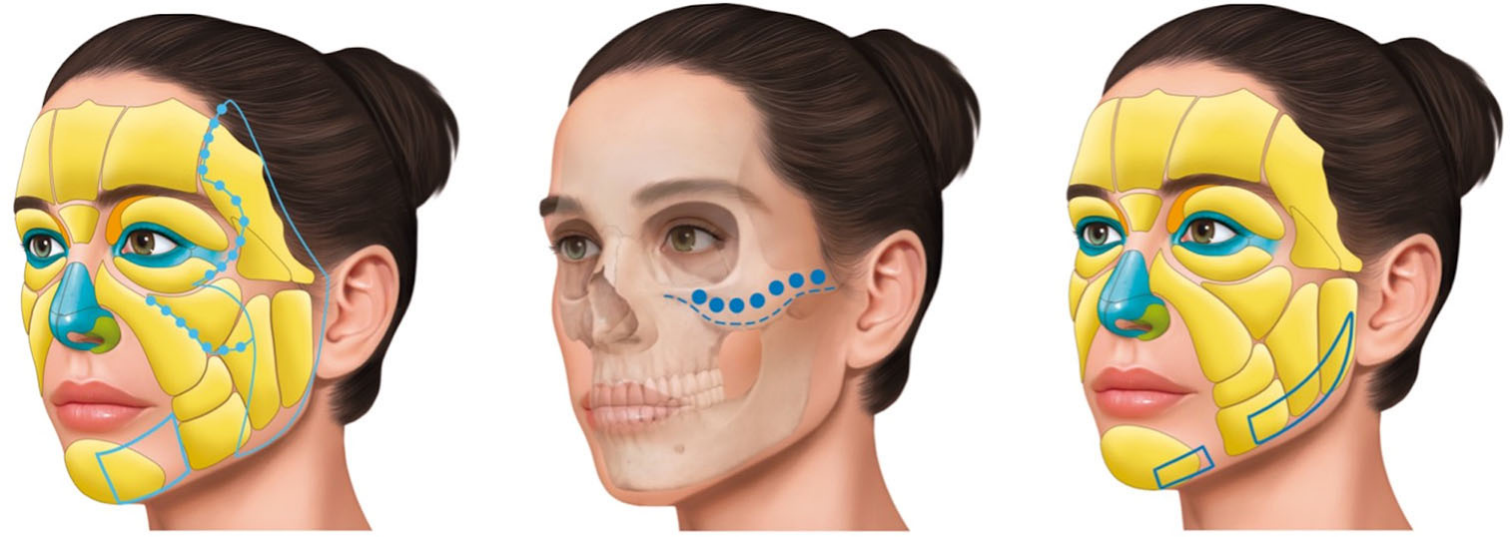
Injection areas in the subcutaneous and supraperiosteal facial planes. 1: Radiesse Duo injection areas in the subcutaneous plane aim to create a vector to lift the face. 2: Radiesse Lidocaine injection areas in the supraperiosteal plane. 3: Radiesse Lidocaine injection areas in the subcutaneous plane

Participants were clinically evaluated for skin laxity, ptosis of subcutaneous fat, and shadow lines correlated with ptosis of ligaments. Injection of different CaHA formulations (Radiesse Lidocaine^TM^ and Radiesse Duo^TM^) was carried out according to the need for facial restructuring: Radiesse Lidocaine^TM^ was used in the subSMAS layer, Radiesse Duo^TM^ was administered to superficial areas, while a combination of Radiesse Lidocaine^TM^ and Radiesse Duo^TM^ was used for restructuring of facial ligaments (Fig. 1).

All participants of this study were administered a total of two syringes each of Radiesse Lidocaine^TM^ and two syringes each of Radiesse Duo^TM^, diluted with 0.5 mL of 2% lidocaine, by following the proposed 6-step technique utilizing microcannulas.

### The 6-Step Technique

#### Step 1: Markings and Delimitations of the Regions for Treatment

We delimited the following areas of medial superior injection: temporal region (temporal line to upper edge of the zygomatic arch); zygomatic region (zygomatic arch); masseteric parotid region (lower edge of the zygomatic arch to a rectangular area of 2–3 cm in the masseteric parotid region); and the ligament transition areas (infraorbital and zygomatic/malar areas).

**Fig. 2:**
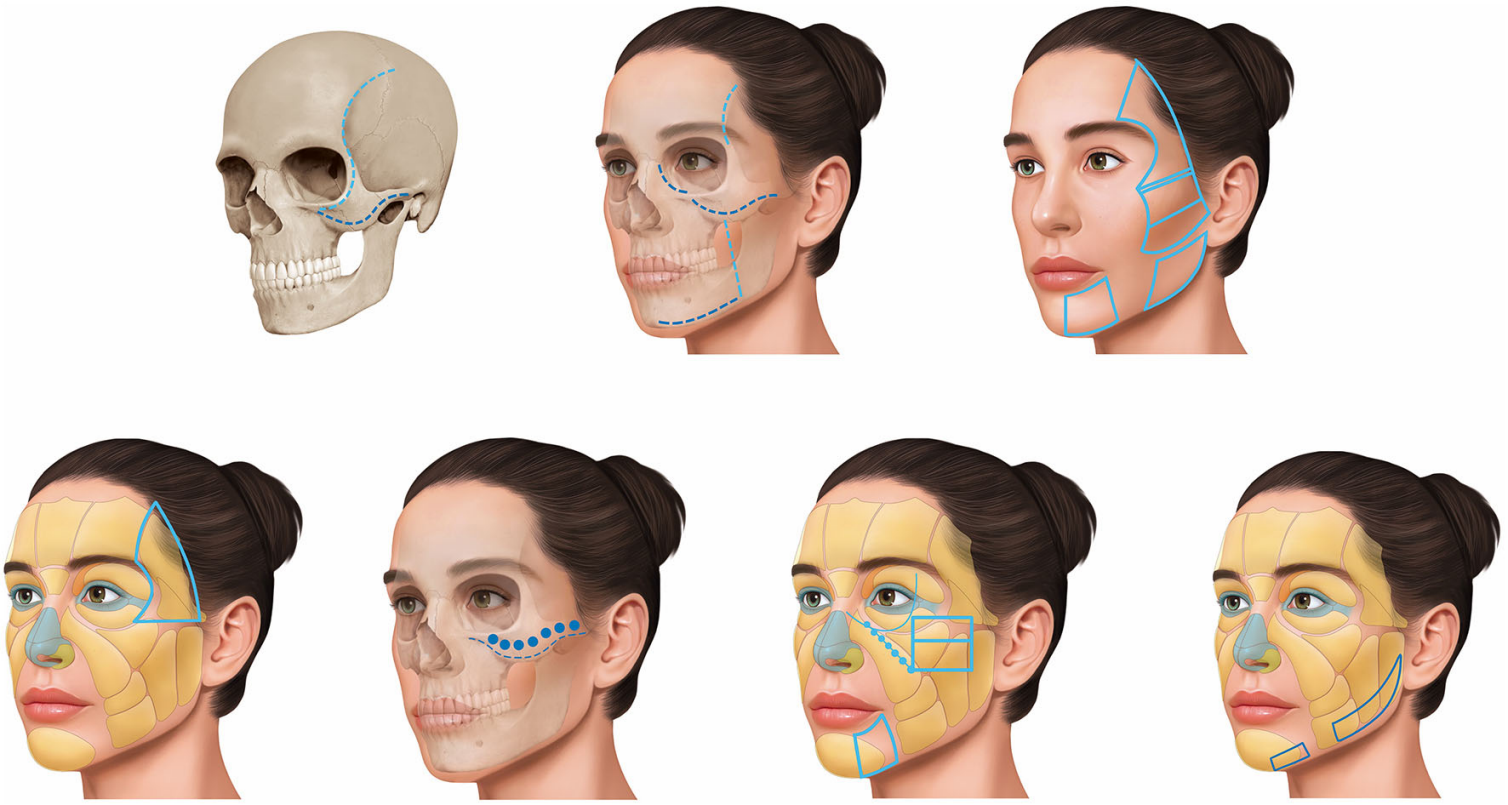
Facial mapping for a series of injections. Top line: Markings and delimitations of the regions for treatment. Bottom line: The injection sequence comprising Radiesse Duo (first syringe), Radiesse Lidocaine (second syringe), Radiesse Duo (third syringe), and Radiesse Lidocaine (fourth syringe)

We delimited the following lower injections areas: the mandible angle region; the pre-jowl regions; and the planes of injection (Fig. 2).

#### Step 2: Solution Preparation

The contents (1.5 mL) of a single Radiesse Duo^TM^ syringe were mixed with 0.5 mL of 2% lidocaine by employing a direct connector or a female-to-female Luer-lock connector, resulting in a total volume of 2 mL per syringe.

#### Step 3: Injection Technique for the First Syringe of Radiesse Duo^TM^

The first access point was located immediately lateral to the transition region, fixed face to mobile face, at the zygomatic region for treatment of the temporal region, and refinement in the infraorbital region.

To treat the temporal region, we injected 0.8 mL of the solution per side using a 23G blunt cannula to cover the entire area. Employing retrograde linear injection or retrograde fanning techniques within the subcutaneous superficial layer, we proceeded from the posterior boundary to the orbital margin, targeting the temporal adhesion ligament and the upper edge of the zygomatic arch.

For the treatment and enhancement of the ligament adhesion regions (temporal ligament adhesion region and from the lateral orbital adhesion to the lateral orbital thickening), as well as the infraorbital area (orbicular retaining ligament), we utilized the same entry point. We injected 0.2 mL of the solution per side using a 23G blunt cannula, employing multiple retrograde micro boluses (Fig. 1). It is advisable to gently massage the treated area immediately after the injection regimen to ensure uniform CaHa distribution.

#### Step 4: Injection Technique for the First Syringe of Radiesse Lidocaine^TM^

For treating the zygomatic region, the same entry point was employed as mentioned above. A volume of 0.45 mL of Radiesse Lidocaine^TM^ per side was injected using a 23G blunt cannula along the juxtaperiosteal plane (subSMAS), encompassing the entire zygomatic area. Additionally, 0.3 mL per side was injected at the malar region, following a juxtaperiosteal plane (around the zygomatic cutaneous ligament), employing a retrograde linear injection technique with micro boluses in the deep layer (Fig. 1). A gentle massage of the treated area immediately post-injection is recommended to ensure a uniform CaHa distribution.

#### Step 5: Injection Technique for the Second Syringe of Radiesse Duo^TM^

The second entry point was established just lateral to the region of transition between the fixed and mobile aspects of the face and was positioned approximately 3 cm beneath the lower border of the zygomatic arch. This access point is employed for treating the masseteric parotid region as well as the ligament transition areas (infraorbital and the zygomatic/malar region).

A second access point was utilized to treat the masseteric parotid region and involved the injection of 0.6 mL of the solution per side using a 23G blunt cannula, employing retrograde linear injection or retrograde fanning techniques. A gentle massage of the treated area immediately post-injection ensured a uniform CaHa distribution.

For the refinement of the ligament transition areas (infraorbital and zygomatic/malar regions), the same access point as above was employed, to administer 0.1 mL of the solution per side with a 23G blunt cannula, utilizing multiple retrograde micro boluses. As before, a gentle massage of the treated area immediately following the injection regimen aids in an even CaHa distribution.

The third access point was positioned lateral to the mandibular cutaneous ligaments to treat the pre-jowl regions and mandibular angle regions.

For the treatment of the pre-jowl region within the mental sulcus, the third access point is employed. This involves injecting 0.3 mL of the solution per side using a 23G blunt cannula, utilizing anterograde and/or retrograde linear injection, or anterograde and/or retrograde fanning techniques at the subcutaneous layer. A gentle massaging of the treated area immediately post-injection ensured an even CaHa distribution.

#### Step 6: The Injection Technique for the Second Syringe of Radiesse Lidocaine^TM^

For an improved outcome, the lower face can be treated with a focus on repositioning the mandibular ligament through interventions in both the pre-jowl and lateral mandible regions.

To administer the mandible treatment, the same access point as mentioned above was employed. We injected 0.45 mL of the solution per side at the lateral mandible region and 0.3 mL per side at the pre-jowl region using a 23G blunt cannula. We employed anterograde and/or retrograde linear injection or anterograde and/or retrograde fanning techniques. Following the procedure, a gentle massage of the treated area ensures even CaHa distribution.

## Results

The 36 patients reported subjective improvement in skin quality and repositioning of the midface at the 90-day evaluation. Twenty-eight experienced swelling in the first 48 hours, and they were advised to enhance post-procedure facial massage, leading to spontaneous symptom relief. Four patients developed swelling after 48 hours and were instructed to use prednisone 20 mg for 3 days, resulting in symptom remission. Three patients complained of persistent pain in the malar region for 20 days and were advised to use analgesics for symptom control. Two patients developed small nodules due to product accumulation, which resolved after intranodular saline injection followed by local massage. No infectious or vascular adverse effects were observed.

Herein, we present cases that illustrate the use of this approach.

**Fig. 3:**
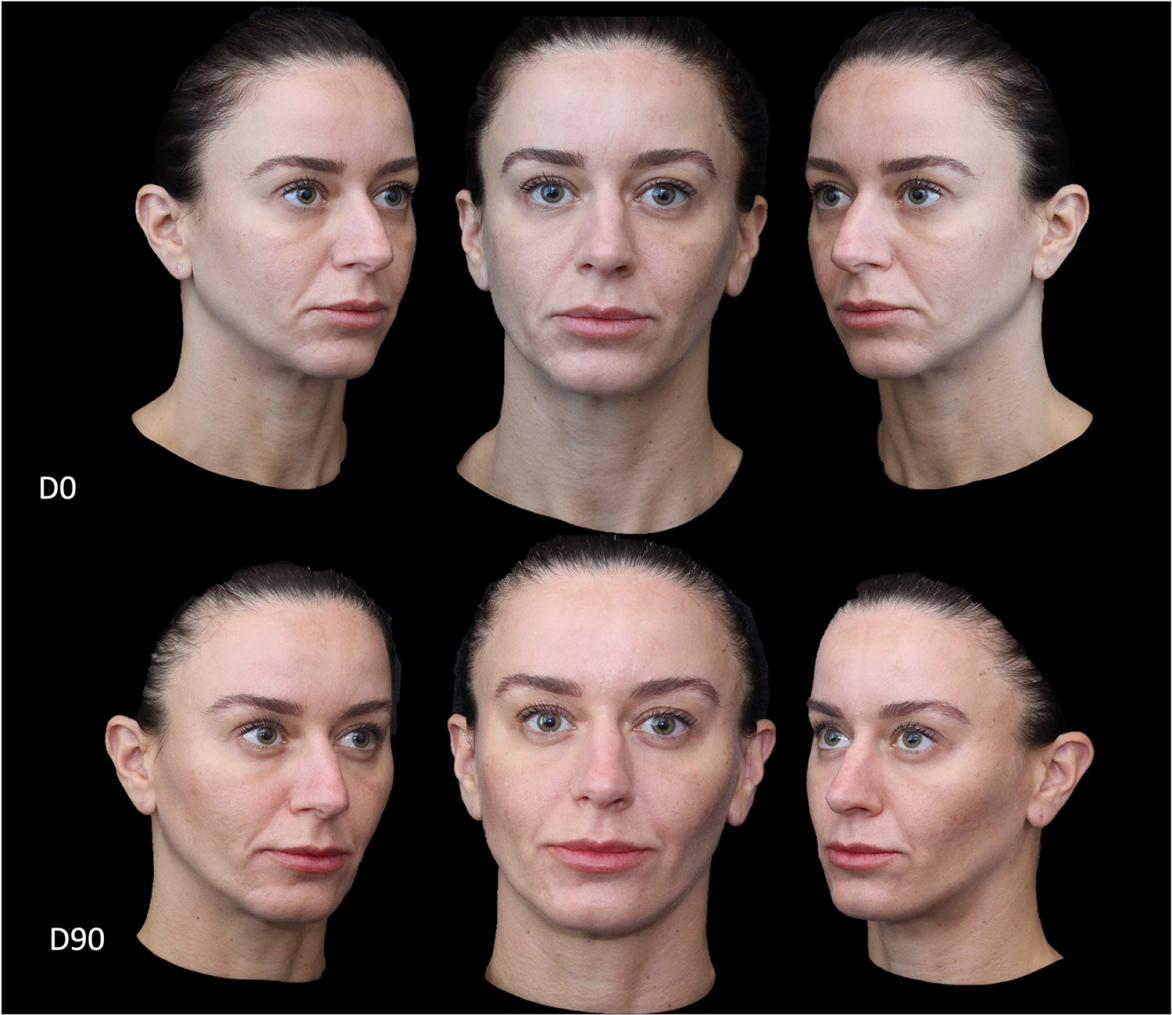
Timeline of treatment comprising Day 0 (D0; day of treatment) and Day 90 (D90; 90 days after treatment) time-points for a patient who underwent the V-lift technique. A generalized improvement of the shadows of the face, restoration of the zygomatic arch and malar region, attenuation of nasolabial lines, and elevation of the angle of the mouth can be observed 90 days after completion of the V-lift technique

**Fig. 4:**
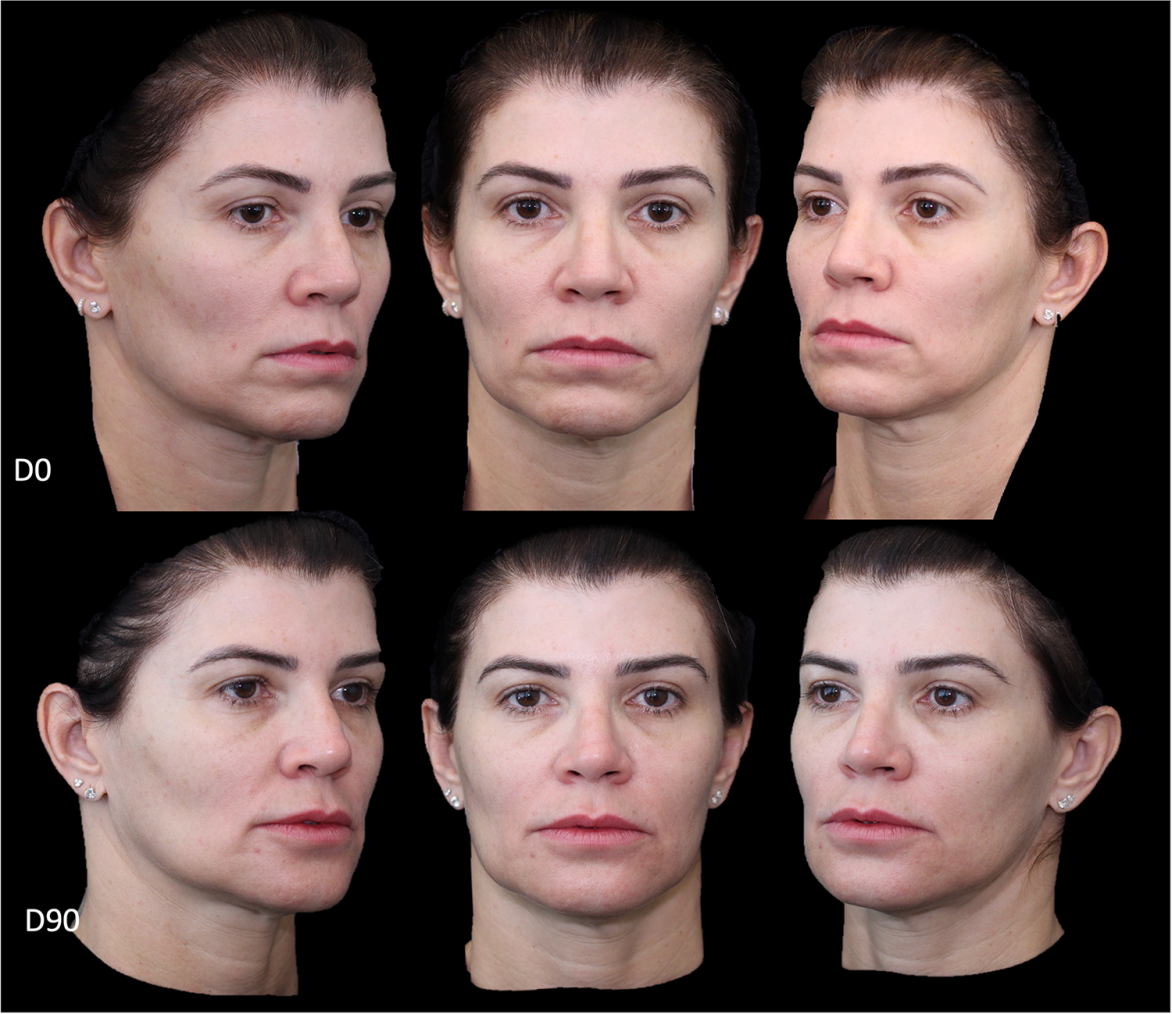
Timeline of treatment comprising Day 0 (D0; day of treatment) and Day 90 (D90; 90 days after treatment) time-points for a patient who underwent the V-lift technique. A generalized improvement of face shadows, restoration of the zygomatic arch, attenuation of the malar mound with restoration of the malar region, attenuation of nasolabial lines, and elevation of the pre-jowl area can be observed 90 days after completion of the V-lift technique

**Fig. 5:**
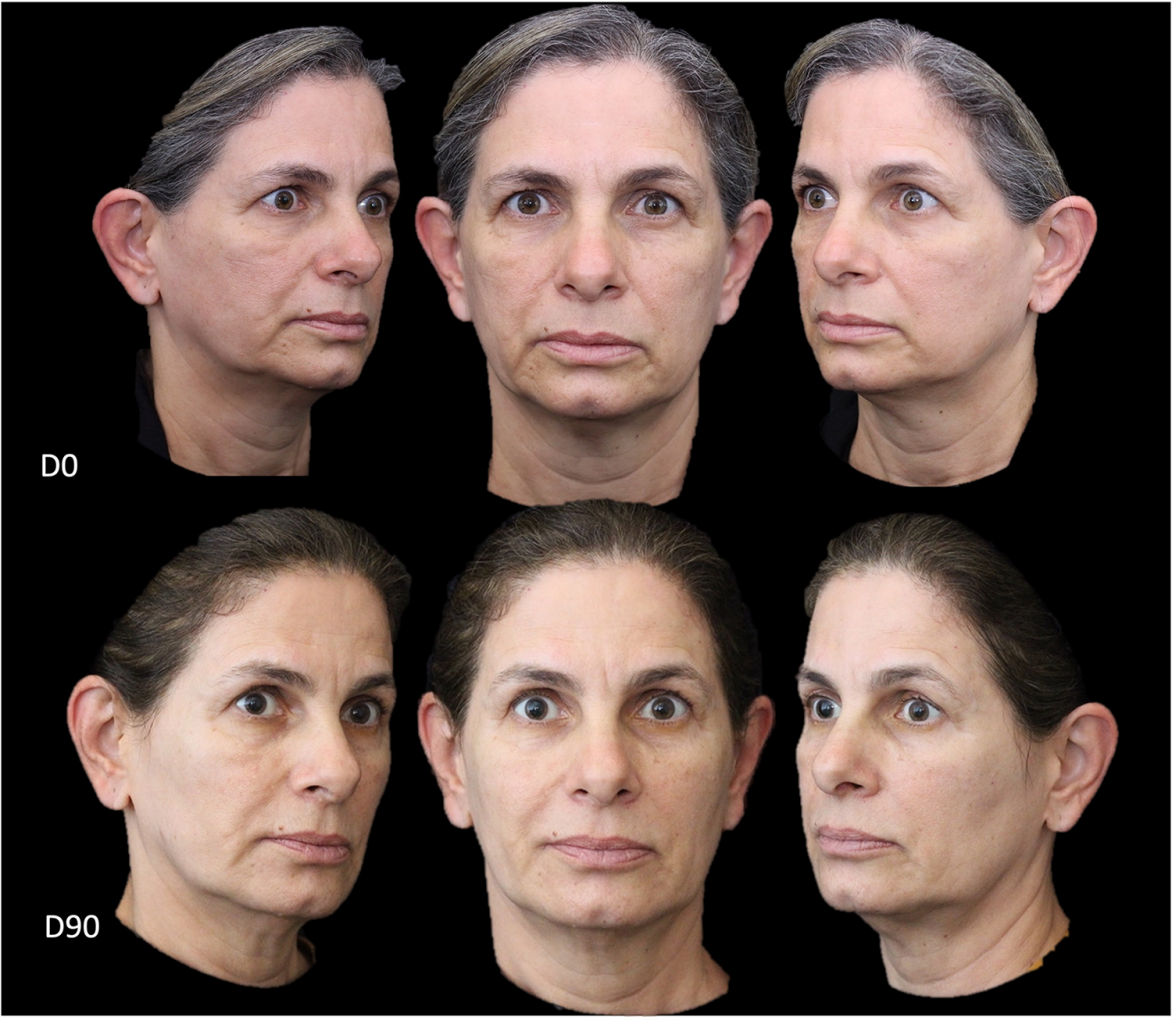
Timeline of treatment comprising Day 0 (D0; day of treatment) and Day 90 (D90; 90 days after treatment) time-points for a patient who underwent a V-lift technique. Note the global repositioning of tissues with the resulting softer facial expression

**Fig. 6:**
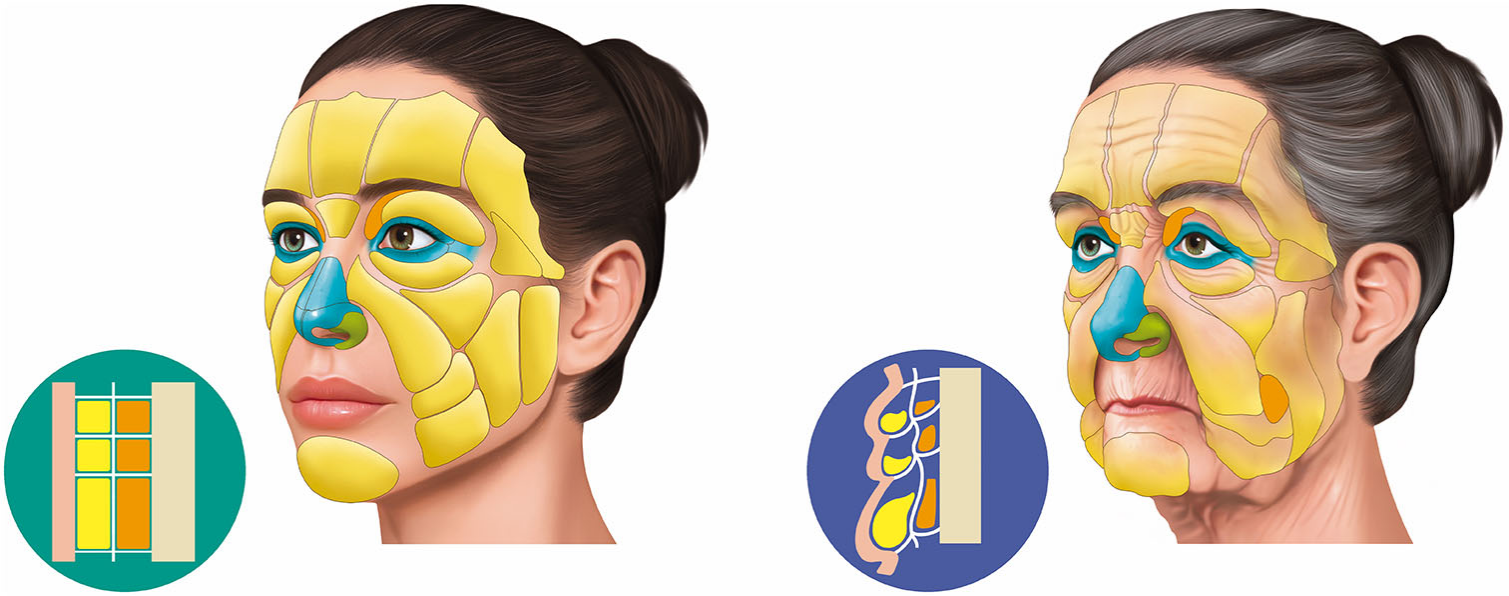
A comparison of a young and aging face showing the three-dimensional aging process. Left: A schematic representation of the face of a young woman with well positioned anatomical layers. Right: A schematic representation of an aging face with changes in the positioning of ligaments due to the gravitational weight of anatomical compartments determined by aging

**Fig. 7:**
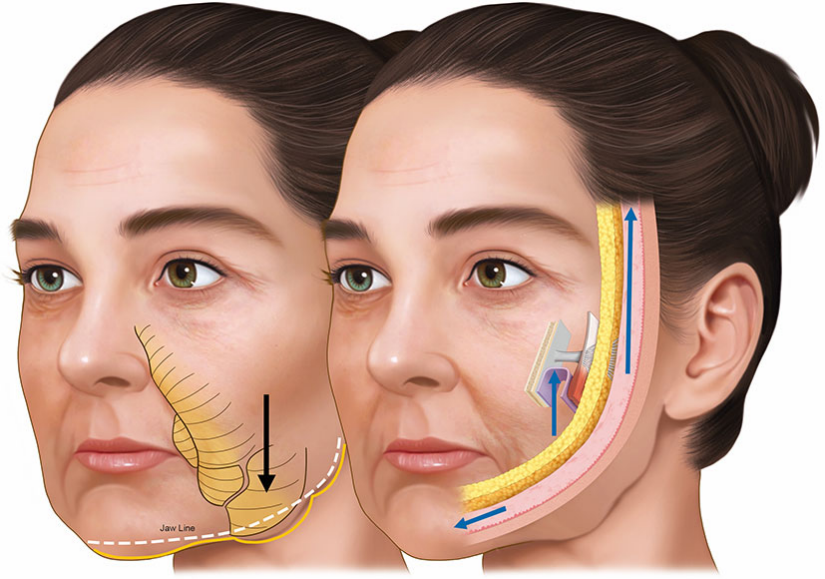
A schematic representation of the repositioning of the cutaneous ligaments leading to a suspension effect

The overall improvements in facial laxity and contour 90 days after the V-Lift procedure are presented in Figs. 3, 4, 5, 6, and 7.

Case 1 (Fig. 3) involved a woman with complaints of facial laxity and loss of facial contour. Ninety days after the 6-step V-lift injection regimen, it was possible to note restoration of the global positioning of the facial tissues with better positioning of the eyebrow, enhancement of the zygomatic malar layers redensification points and attenuation of the nasolabial, mental and labiomental folds with significant elevation of the lip commissure.

In Case 2 (Fig. 4), we present a lady with a “heavy” face, with a saggy appearance of the whole face and deep markings at the malar region. Ninety days after the V-lift injections, it was possible to discern attenuation of the grooves and amelioration of global face complaints.

In Case 3 (Fig. 5), we present a woman with skin laxity, loss of facial contour and a “sad mouth” appearance. Ninety days after the V-lift procedure, a restoration of the global positioning of the face with better positioning of the eyebrows was observed, as well as an enhancement of the zygomatic malar layers redensification points, attenuation of the nasolabial, mental, and labiomental folds, and a significant elevation of the lip commissure.

## Discussion

The depletion of anatomical tissues is a consequence of the aging process observed throughout the layers of facial soft tissue (Fig. 6), and in addition to skeletal changes [2, 10–14].

The role of the retaining ligaments in the process of facial aging is not well defined [10]. Some authors believe that the loosening of the retaining ligaments results in the descent of the soft tissue that they normally support [12, 15, 16]. Other groups postulate that ligaments retain their relative strength, while the surrounding tissues experience a loss of support and subsequently descend over these ligaments that remain relatively stable in their position, leading to the formation of grooves [7, 17, 18].

In any case, age-related alterations in facial bone and skin are widely recognized. The ongoing and lifelong remodeling of the bony framework, coupled with the loosening of the skin, leads to the distortion of ligament attachment points and their adhesions to the skin. This, in turn, impacts the neighboring structures supported by these ligaments, as the changed position and trajectory of the ligaments create a ripple effect [19, 20].

**Fig. 8:**
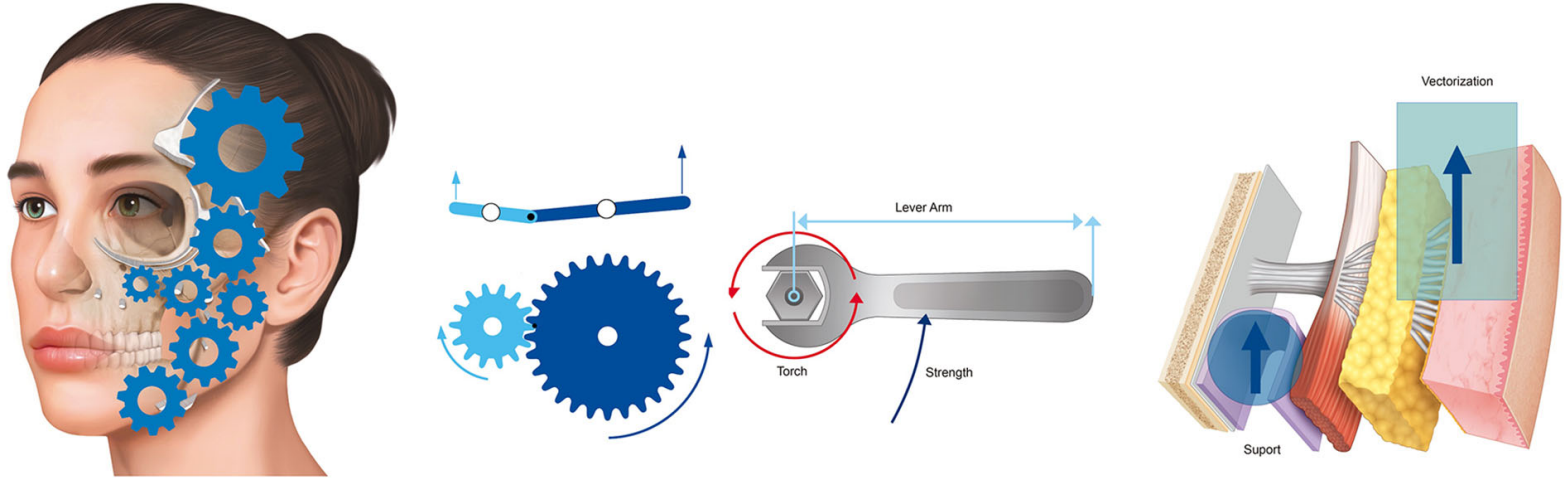
Biomechanical subMAS layer redensification mechanisms and vectorization of the facial ligaments implement the global repositioning of the face as a mechanism of pulleys interconnected by the superficial musculoaponeurotic system (SMAS) and superficial temporal fascia

For these reasons, approaches that focus exclusively on facial volume restoration often yield esthetically unsatisfactory outcomes because they require larger additional volumes to achieve noticeable results in the superficial layer [21]. Cong et al. [5] described a method that differs from the augmentation of facial fat compartments in that it was not intended to restore the volume of the deflated fat compartments, but to achieve a vertical tissue displacement, that is, a lifting effect. Our technique is based on the concept that injecting CaHa around the regions of adhesion and retaining ligaments of the face triggers the repositioning of the cutaneous ligaments, resulting in a suspension effect. These ligaments effectively serve as anchoring points for the SMAS and the dermis, overlying the deep fascia and periosteum (Fig. 8) [8]. The role of CaHa in improving the mechanical characteristics of the skin has been broadly reported, as it stimulates collagen and elastin production, angiogenesis, and dermal cell proliferation [8] that could be an advantage of the present technique, as it could offer layers redensification.

**Fig. 9:**
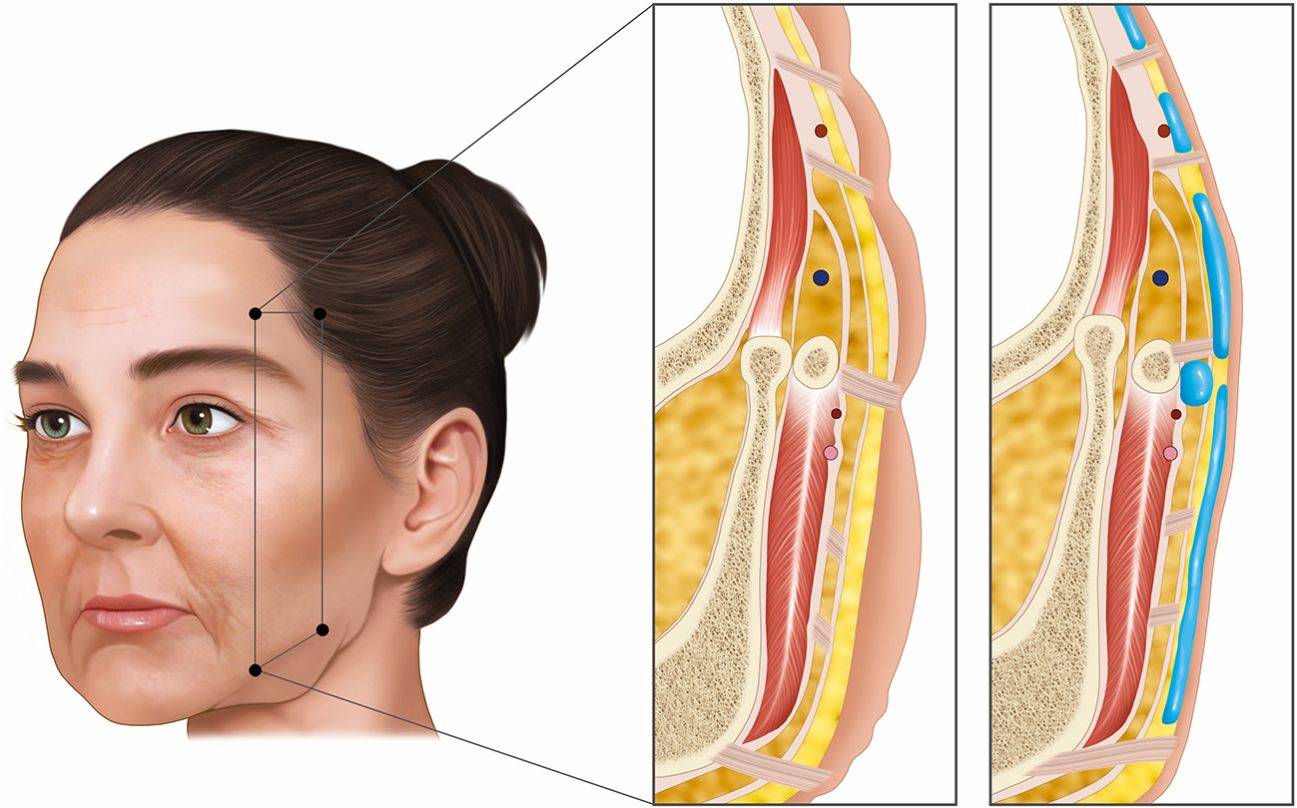
A schematic representation of the planes of injection, vectors and direction of injection of different types of CaHA, according to study objectives and definition of facial planes

The facial retaining ligaments are robust, deep, and fibrous connections that stem from the periosteum or the deep facial fascia. These ligaments traverse facial layers perpendicularly, anchoring into the dermis. Attenuation of the retaining ligaments at all levels reduces the quality of fixation of the layers (5). Hence, three-dimensional facial repositioning interventions encompass the vertical adjustment of these ligaments. This approach directly counteracts the detrimental impact of negative weight vectors generated by the aging process on the face [22]. This concept can be elucidated through the “torch” theory, where the robustness of CaHA collagen biostimulator enables the elevation of facial structures (Fig. 9). Movement/Moment/Torque is a mathematical variable that scales the direction of movement based on the balance of forces. Conditioned by the vectors that depress or elevate the face, the ligaments must be evaluated as lever arms that have a fixed origin (bone) and a mobile insertion point (skin). It is necessary to consider the ligament as a lever arm, its position being directly influenced by deep repositioning (subMAS layer redensification—without distension/enlargement of the compartment) and superficial repositioning (vectoring with gain in skin quality) through the superficial suspension force of the SMAS, considering the zygomatic arch as the bone support point. Thus, the positive (counterclockwise) direction of the torque was directly influenced by the vector force and the angle related to the origin or axial point of rotation (Fig. 9).

**Fig. 10:**
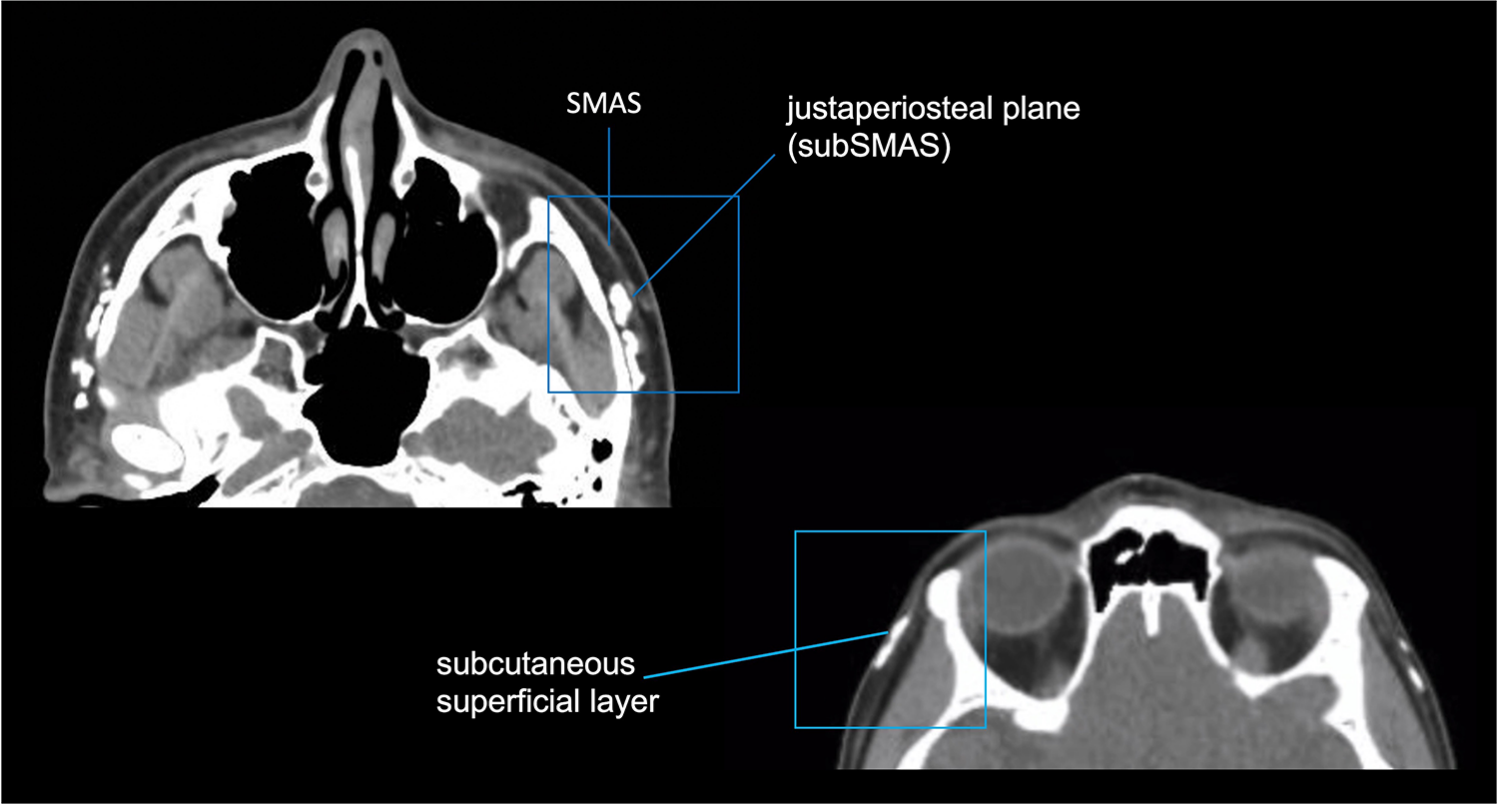
Face CT scan showing CaHa injection in the zygomatic arch in the subSMAS plane (justaperiosteal) and in the temporal region in the subcutaneous plane

The objective of the technique is to address the ligamentous origin through the redensification of underlying tissues by injecting calcium hydroxylapatite (CaHa) in the justaperiosteal plane (subSMAS). In the subdermal plane, the goal is the potential stretching of superficial ligamentous fibers through the supposed vectorization implemented by neocollagenesis. Therefore, the focus is not on the ligament itself as a specific target, but rather on its territory. While the study did not include imaging examinations for all patients, we had the opportunity to assess the injection planes in a patient who underwent a CT scan for another reason (Fig. 10), 21 days after CaHa injection in the midface and bilateral temples. Since CaHa is radiopaque, the injection plane of the product could be visualized according to the described technique.

For this reason, we consider the Radiesse line to be a valuable treatment instrument for the profile of patients who aim for three-dimensional face repositioning and progressive results without subsequent volumetric gains, as there is less hydrophilic effect compared to hyaluronic acid; thus, a stable overall esthetic outcome is attained. Only a small volume is needed for this ligament injection technique, and the 23G blunt cannula was chosen due to the possibility of accessing the desired layers (supraperiosteal and subdermic).

Site 1 was placed in the temporal ligamentous adhesion region to lift the tail of the eyebrow, and site 2 was placed in the region of the lateral orbital thickening to reduce ptosis of the lateral canthus. Injections at the two sites had a lifting effect on the periorbital region, as early ptosis of the eyebrows and lateral canthi could be partly reversed in a subtle manner, restoring a youthful appearance. As for the midface, site 3 and site 4 targeted the zygomatic retaining ligament and zygomatic cutaneous ligament, respectively, to augment the midfacial soft tissue. The apex of the cheekbone intensely reflected light, and the length of the lower eyelid seemed to decrease due to the interplay of light and shadow, contributing to a visual lifting effect. CaHa injections at these two sites would also affect the soft tissue of the anteromedial midface, so that the nasolabial fold would be simultaneously effaced. In the lower face, the nasolabial fold and the pre-jowl sulcus were also reduced by the injections at sites 5 and 6, corresponding to the regions of the maxillary and mandibular ligaments, respectively. Therefore, injecting at the sites corresponding to the actual ligament areas could effectively enhance the redensification effect of the subSMAS layer, simultaneously reducing facial grooves and alleviating the signs of aging. Upon completion of the treatment, which involves lifting the upper and middle face, significant enhancements were noted in the infraorbital hollow and the repositioning of the overall facial volume. Moreover, this treatment has the ability to restore volume, tissue resilience, and harmony within the modiolus region, marionette zone, and pre-jowl sulcus. The main point of this technique involves attaining natural outcomes following facial procedures by using less volume and shortened distances. These natural-looking results play a pivotal role in enhancing patient satisfaction and bolstering self-assurance as natural outcomes have the potential to alleviate the stigma often associated with cosmetic procedures [22–24].

This study has certain limitations. It is based on subjective observations made by the authors, a factor that warrants consideration when interpreting the presented data. Future investigations should explore the histological and mechanical characteristics of the ligaments following treatment with collagen biostimulators. This could be achieved by incorporating a control group, employing randomized selection, and utilizing objective outcome measures. Furthermore, studies encompassing a broader participant pool and a prolonged postoperative timeline are imperative for a more comprehensive understanding. A prospective study is warranted to quantify the improvement in facial aging achieved through the Radiesse V-Lift technique. This study should incorporate control groups, implement blinding methodologies, and employ objective outcome measures.

## Conclusion

We present the preliminary results of a three-dimensional injection technique known as the V-lift technique, which utilizes calcium hydroxylapatite (CaHA) collagen biostimulators to address the signs of aging. The V-lift technique was well-tolerated, yielding enduring subjective results attributed to the stimulation of collagen and elastic fiber deposition (collagenesis). However, more robust studies are needed to substantiate these concepts.
