# Correction: An Innovative Treatment Using Calcium Hydroxyapatite for Non-Surgical Facial Rejuvenation: The Vectorial-Lift Technique

**DOI:** 10.1007/s00266-024-04167-y

**Published:** 2024-06-13

**Authors:** Virginia Marcia Amaral, Helena Hotz Arroyo Ramos, Fernanda Aquino Cavallieri, Mariana Muniz, Guilherme Muzy, Ada Trindade de Almeida

**Affiliations:** 1 IVA Medical Institute, Av. dos Bandeirantes 1518, Belo Horizonte, CEP: 30.315-032 Brazil; 2 Vitória, Brazil; 3 Rio de Janeiro, Brazil; 4 São Paulo, Brazil; 5 São Paulo, Brazil; 6 Hospital do Servidor Público Municipal de São Paulo, São Paulo, Brazil

**Correction to: Aesth Plast Surg** 10.1007/s00266-024-04071-5

The article “An Innovative Treatment Using Calcium Hydroxyapatite for Non-Surgical Facial Rejuvenation: The Vectorial-Lift Technique,” written by Virginia Marcia Amaral et al, was originally published electronically on the publisher’s internet portal on 7 May 2024 without open access. With the author(s)’ decision to opt for Open Choice the copyright of the article changed on 23 May 2024 to © The Author 2024 and the article is forthwith distributed under a Creative Commons Attribution 4.0 International License, which permits use, sharing, adaptation, distribution and reproduction in any medium or format, as long as you give appropriate credit to the original author(s) and the source, provide a link to the Creative Commons licence, and indicate if changes were made. The images or other third party material in this article are included in the article’s Creative Commons licence, unless indicated otherwise in a credit line to the material. If material is not included in the article’s Creative Commons licence and your intended use is not permitted by statutory regulation or exceeds the permitted use, you will need to obtain permission directly from the copyright holder. To view a copy of this licence, visit http://creativecommons.org/licenses/by/4.0/.

